# From Fragile Enamel to Functional Harmony: A Case Report of the Twin-Stage Approach to Amelogenesis Imperfecta

**DOI:** 10.7759/cureus.108185

**Published:** 2026-05-03

**Authors:** Bhavani Ch, Sreeramulu Basapogu, Swapna Basimi, Pilli Bhavana, Akhila Donthoji, Sri Manasa Boge

**Affiliations:** 1 Prosthodontics, Government Dental College and Hospital, Hyderabad, Hyderabad, IND

**Keywords:** amelogenesis imperfecta, full mouth rehabilitation, hobo twin stage, porcelain fused to metal, vertical dimension of occlusion

## Abstract

Amelogenesis imperfecta (AI) is a hereditary enamel defect that leads to compromised esthetics, tooth sensitivity, and functional impairment. The case report describes the rehabilitation of a 24-year-old male patient diagnosed with hypoplastic AI associated with reduced vertical dimension of occlusion (VDO). A systematic treatment approach was adopted, including diagnostic evaluation, occlusal splint therapy to assess adaptability to increased VDO, and definitive prosthetic rehabilitation using the Hobo twin-stage technique. The VDO was increased by 2 mm to restore functional harmony and provide adequate restorative space. Porcelain-fused-to-metal restorations were fabricated following controlled occlusal principles with canine-guided occlusion. Post-treatment evaluation demonstrated significant improvement in mastication, esthetics, and patient comfort. The Hobo twin-stage technique proved to be a predictable and efficient method for achieving stable occlusion without complex mandibular recordings.

## Introduction

Amelogenesis imperfecta (AI) is a rare genetic condition that affects the formation of dental hard tissue of enamel, resulting in structural and functional defects of teeth [1,2]. These defects may be quantitative or qualitative, depending on the type of AI. In 1988, Witkop proposed a revised classification of AI, considering the nature of the enamel defects as well as the mode of inheritance and dividing AI into four classes (Type I hypoplastic, Type II hypomaturation, Type III hypocalcified, Type IV hypomaturation/hypoplastic with taurodontism) [3,4].

Clinically, AI presents with enamel thinning, discoloration, hypersensitivity, and rapid attrition, often leading to dentin exposure and compromised esthetics [5]. Progressive tooth wear can result in reduced vertical dimension of occlusion (VDO), affecting masticatory efficiency and facial profile. Loss of vertical dimension of occlusion leads to reduced lower facial height, Temporomandibular disorders, and masticatory muscle discomfort.

Full-mouth rehabilitation in such patients requires careful planning and selection of an appropriate occlusal philosophy. Various popular occlusal philosophies and concepts, including the Gnathological concept, Pankey-Mann-Schuyler (PMS) concept [6], Hobo’s concept [7,8], the Biological Occlusion concept, Youdeli’s concept [9], and the Nyman and Lindhe concept. Among these, the PMS philosophy and Hobo’s twin-stage technique are widely used. In cases with relatively normal tooth alignment, Hobo’s twin-stage technique is particularly advantageous. In this approach, occlusal morphology is established based on predetermined standard cusp angles, thereby eliminating the need to record individual condylar guidance. The anterior guidance is then developed in harmony with esthetic, phonetic, functional, and patient comfort requirements. However, a limitation of this technique is that it is not suitable for cases with significant malocclusion. The Hobo twin-stage technique offers advantages in cases with relatively normal alignment, as it eliminates the need for recording condylar guidance and allows standardized occlusal control.

This case report describes the prosthetic full-mouth rehabilitation of a patient with hypoplastic AI using the Hobo twin-stage occlusal scheme.

## Case presentation

A 24-year-old male patient reported to the Department of Prosthodontics with concerns of generalized tooth wear, discoloration (Figure 1), and reduced masticatory efficiency, and complained of sensitivity to cool beverages and difficulty eating food. There was no relevant medical or familial history.

**Figure 1:**
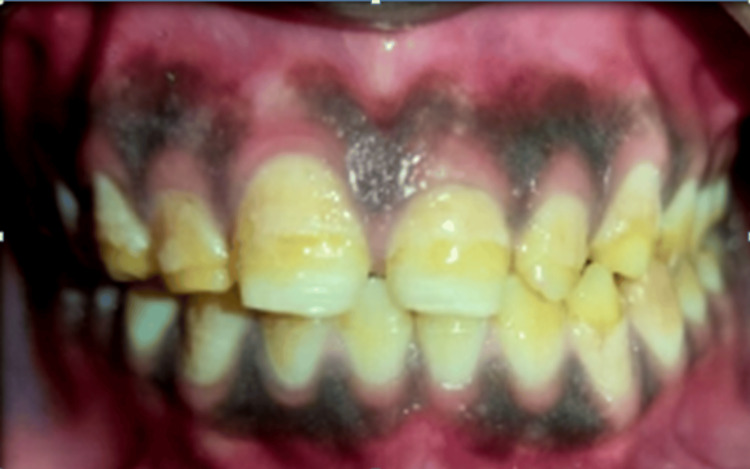
Preoperative view of the amelogenesis imperfecta showing generalized yellowish brown discoloration of teeth with severe occlusal wear in maximum intercuspation.

Extraoral examination revealed normal facial symmetry and musculature (Figure 2). Temporomandibular joint (TMJ) evaluation showed no abnormalities such as pain, clicking, or deviation.

**Figure 2:**
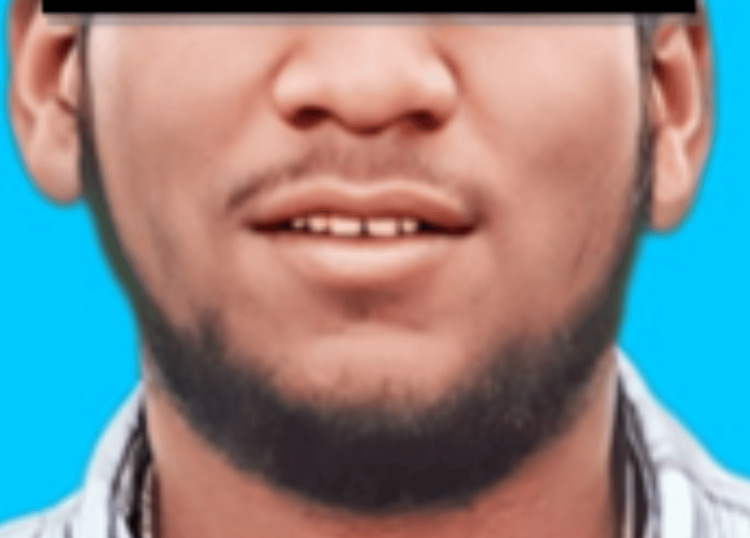
Preoperative extraoral view

Intraoral examination revealed generalized yellowish-brown discoloration and severe occlusal wear with minimal enamel on the occlusal surface (Figure 3). The vertical dimension appeared reduced, with an increased interocclusal freeway space of approximately 6 mm.

**Figure 3:**
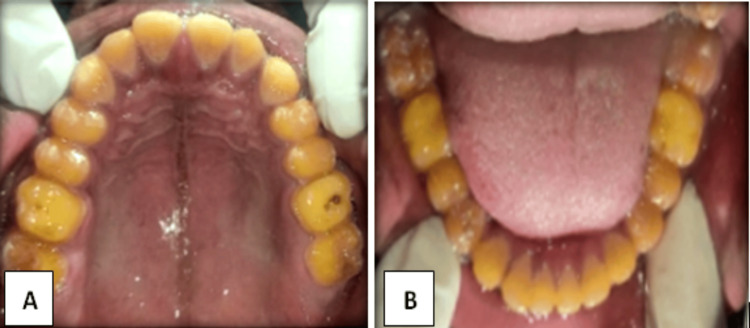
Intra-oral view of maxillary (A) and mandibular (B) arches showing generalized yellowish brown discoloration of teeth with severe occlusal wear

On radiographic examination, there is minimal enamel with well-formed root morphology with closed apices and adequate bone around the teeth. The pulp horns are located 1.5 mm below the occlusal surface (Figure 4).

**Figure 4:**
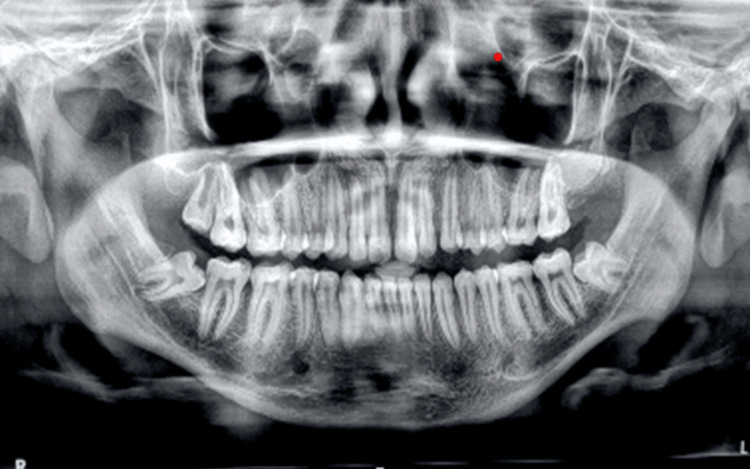
Orthopantomogram showing minimal enamel with well-formed root morphology with closed apices with adequate bone around the teeth; the pulp horns are located 1.5 mm below the occlusal surface

Based on clinical findings, the patient was diagnosed with hypoplastic AI associated with a decreased VDO. A full-mouth rehabilitation was planned using the Hobo twin-stage occlusal scheme.

Pre-prosthetic phase

Diagnostic impressions were made, and maxillary casts were mounted on a semi-adjustable articulator using a face-bow transfer (Figure 5). Centric relation was recorded using bite registration wax material.

**Figure 5:**
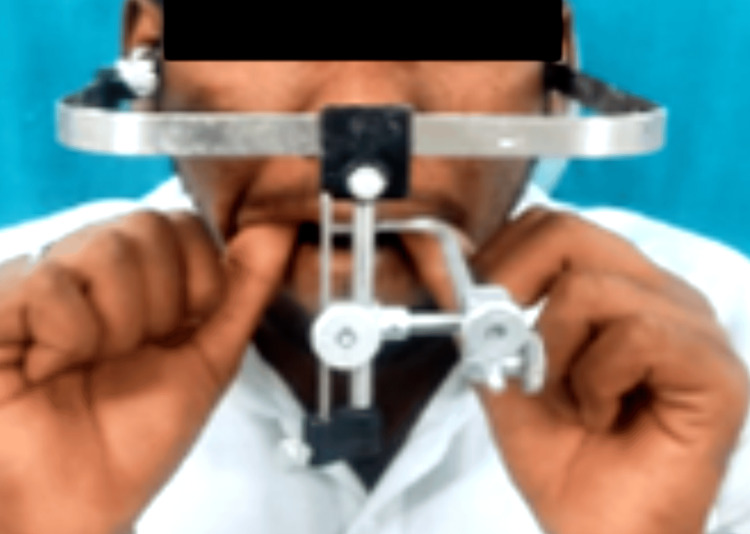
Facebow transfer done to orient the spatial relationship of maxillary arch to their temporomandibular joint and transfer this orientation to an semi-adjustable articulator.

Measurements obtained included (i) Vertical Dimension at Rest (VDR): 68 mm, and (ii) VDO: 62 mm. A decision was made to increase the VDO by 2 mm to achieve a functional interocclusal rest space of approximately 4 mm. An occlusal splint was fabricated and worn by the patient for six weeks to assess adaptability (Figure 6). The patient reported no discomfort, muscle fatigue, or TMJ issues with the raised vertical dimension, which was lost due to an enamel defect.

**Figure 6:**
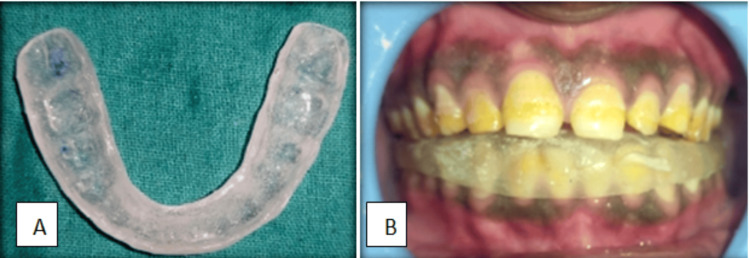
Occlusal splint (A) was applied at increased vertical dimension to permit the entire TMJ complex to attain an improved functional health and a new vertical dimension to be established (B) Occlusal splint in frontal view TMJ:  temporomandibular joint

Increasing the VDO was mandatory to allow the condyles to assume their most superior position in intimate contact with the thinnest portion of the biconcavity of the disc. It permitted the entire TMJ complex to attain an improved functional health and allowed the posterior avoidance patterns of occlusion and a new vertical dimension to be established.

Occlusal Plane Determination

Broadrick’s flag analysis was used to determine the mandibular occlusal plane (Figure 7). A 4-inch radius was used to establish the curve of Spee, ensuring proper occlusal alignment. Its clinical relevance lies in its ability to predict, analyze, and correct deranged occlusal planes, such as tipped, rotated, or extruded teeth, to ensure harmonious function and prevent posterior interferences during protrusion, thus minimizing issues with muscles and the TMJ.

**Figure 7:**
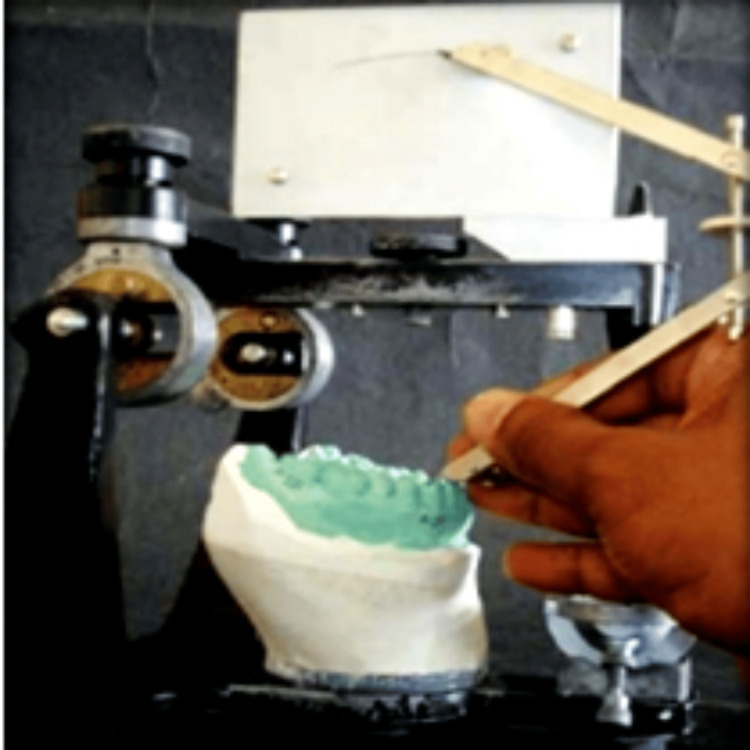
Occlusal plane analysis using broadrick plane analyzer to correct deranged occlusal plane.

Diagnostic Wax-Up

Diagnostic wax-up was done as per standardized values of the Hobo twin-stage technique, which involves 2 stages: condition 1 and condition 2 posterior teeth wax-up, followed by anterior teeth wax-up (Table 1).

**Table 1:** Hobo twin-stage technique standardized values

Condition	Horizontal condylar guidance	Lateral condylar guidance	Anterior guidance	Lateral anterior guidance
Condition 1	25°	15°	25°	10°
Condition 2	40°	15°	45°	20°

Condition 1 (posterior wax-up) included sagittal condylar path (25°), Bennett angle (15°), and lateral wing angle (10°). Condition 2 (used for finalizing anterior wax-up to ensure canine guidance) included sagittal condylar path (40°), Bennett angle (15°), and lateral wing angle (20°) (Figure 8).

**Figure 8:**
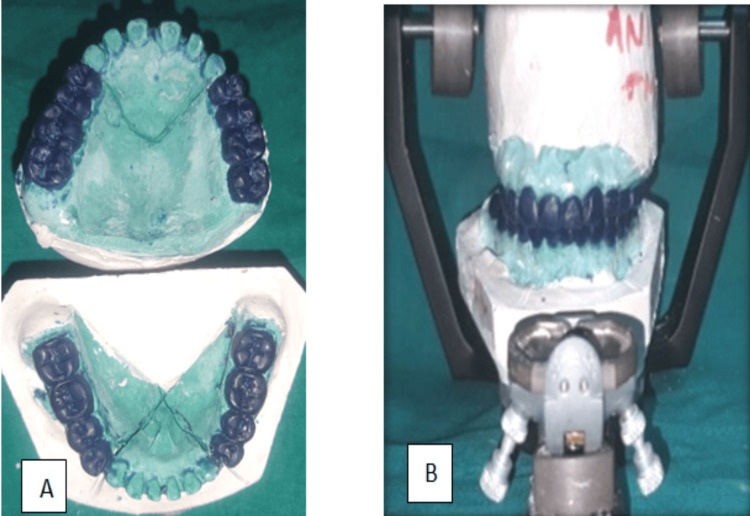
Diagnostic wax up done as per standardized values Hobo twin stage technique. (A) Diagnostic wax-up of posterior teeth done as per condition 1; (B) Diagnostic wax-up of anterior teeth done as per condition 2

Tooth Preparation and Provisionalization

Teeth were prepared using the simultaneous arch technique for full-coverage metal-ceramic restorations with equigingival margins (Figure 9).

**Figure 9:**
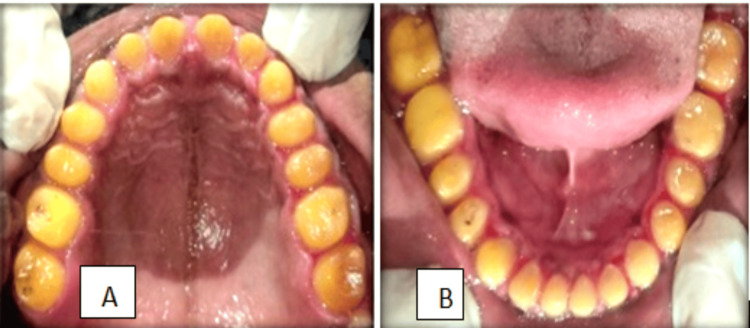
Vital preparation of teeth were done with equigingival margins. (A) Occlusal view of maxillary arch; (B) Occlusal view of mandibular arch

Autopolymerizing acrylic resin provisional crowns were fabricated using putty index on a cast using an indirect technique that was duplicated from the cast with the diagnostic wax-up. The provisional restorations were cemented with temporary cement (Freegenol Temporary Pack; GC Corporation, Tokyo, Japan), and the patient’s adaptation was monitored over a three-month period (Figure 10).

**Figure 10:**
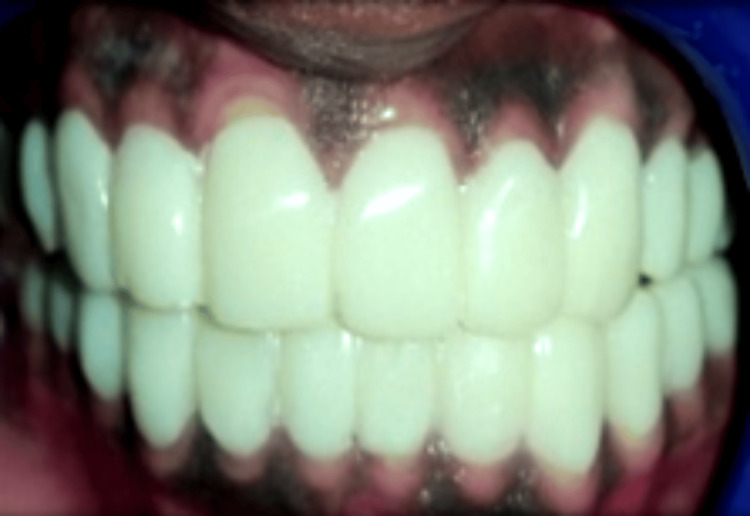
Provisionalization done for the purpose of pulpal protection, maintaining periodontal health ,ensuring positional stability and evaluating esthetic and function before final prosthesis cementation

The provisional restorations also served as a guide for planning the definitive prosthetic rehabilitation. Throughout this phase, functional parameters such as muscle tenderness, TMJ discomfort, masticatory efficiency, mandibular movements, swallowing, and speech were systematically evaluated.

Notable improvements in mastication, speech, and facial esthetics confirmed adaptation to the altered mandibular position with the restored VDO. Bite registration was performed using occlusal registration material by sequentially removing provisionals on the right side of the arch while maintaining the provisionals on the left side of the arch and vice versa.

Definitive Impressions

Definitive impressions were made using polyvinylsiloxane impression material (Figure 11).

**Figure 11:**
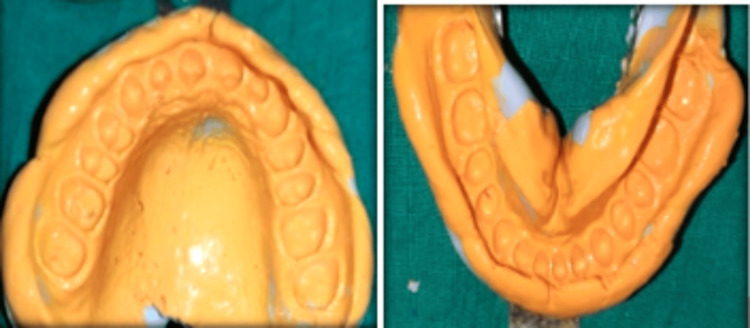
Definitive impressions made with elastomeric impression materials

Prosthetic phase 

After one week, a metal trial was done to evaluate fit, marginal accuracy, proximal contact stability, retention, and occlusion of the cast metal framework before porcelain application (Figure 12).

**Figure 12:**
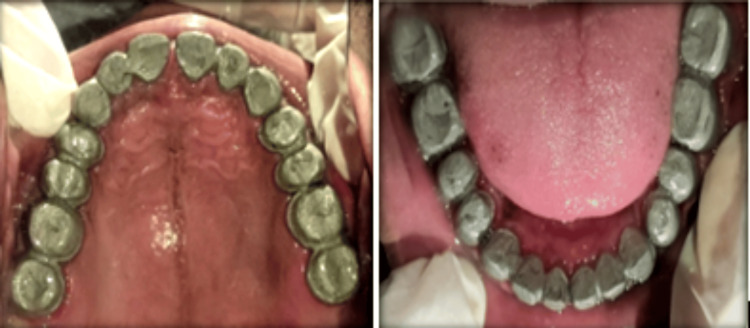
Metal try-in done in maxillary and mandibular arch

Porcelain-fused-to-metal restorations were fabricated using diagnostic wax-up, utilizing the duplicated cast of the provisional restoration and luted with resin-modified glass ionomer cement as quadrant wise in a simultaneous arch form (Figure 13) and the final prosthesis in centric occlusion relation (Figure 14).

**Figure 13:**
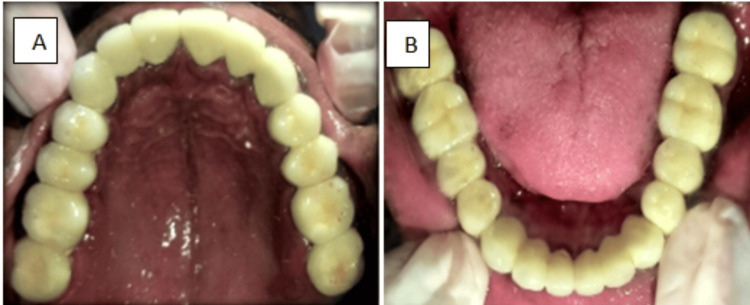
Occlusal view of final prosthesis cemented as quadrant wise in simultaneous arch form (A) Occlusal view of final prosthesis in maxillary arch; (B) Occlusal view of final prosthesis in mandibular arch

**Figure 14:**
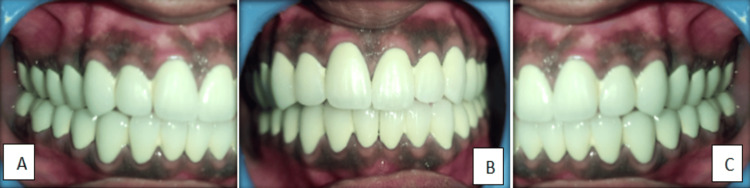
Cemention done with glass ionomer cement (A) Right lateral view; (B) Frontal view; (C) Left lateral view

The final prostheses were fabricated using mutually protected and canine-guided occlusion (Figure 15).

**Figure 15:**
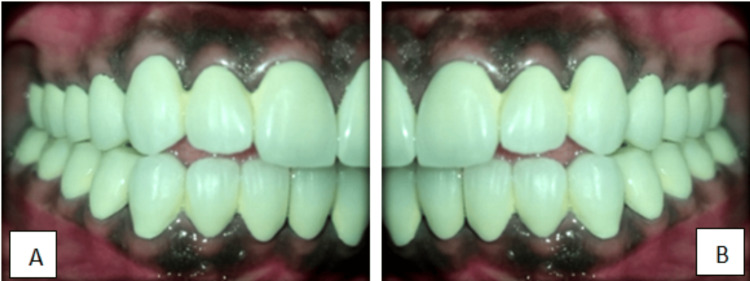
Eccentric view with canine-guided occlusion (A) Right eccentric view; (B) Left eccentric view

The anterior teeth protected the posterior teeth from excessive force and wear, and the posterior teeth supported the occlusal load in Centric occlusion. Oral hygiene instructions were given, and follow-up visits were scheduled at 15 days, one month, three months, six months, and in every recall visit, the patient's comfort, any occlusal discrepancies, and mastication were addressed, and the patient was satisfied with the prosthetic restoration (Figure 16).

**Figure 16:**
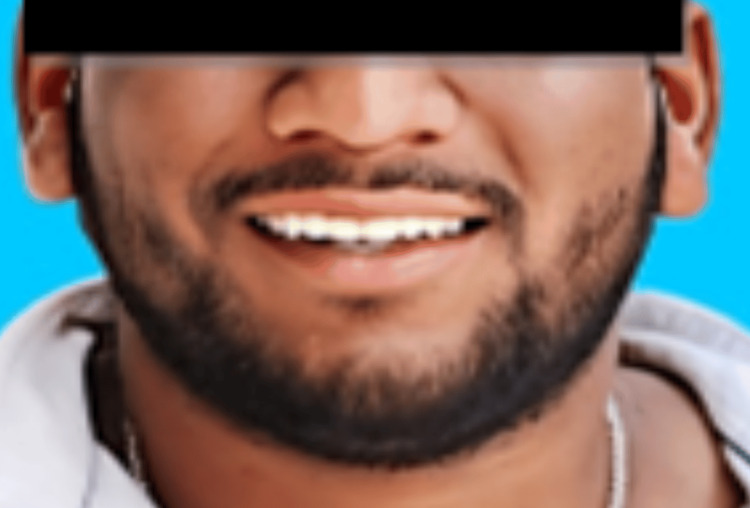
Postoperative view

## Discussion

Treatment of reduced vertical dimension is primarily directed toward restoring the lost vertical dimension rather than increasing it beyond physiological limits. The ultimate objective is to achieve optimal functional efficiency and harmonious coordination among the teeth, muscles, temporomandibular joints, and supporting structures of the stomatognathic system [10].

According to Dawson, interocclusal space is not permanently lost, as the body adapts through compensatory mechanisms such as continuous tooth eruption, alveolar bone remodeling, and neuromuscular adjustments [10]. Both the VDO and the VDR are dynamic parameters that can change over time. Clinically, VDO can be safely increased up to the level of VDR, which represents a physiologic position of minimal muscular activity and serves as a reliable reference for occlusal rehabilitation.

In the present case, the decision to increase the vertical dimension by 2 mm was made to create sufficient interocclusal space for definitive restorations. A mild increase in VDO is generally considered safe and non-detrimental, provided occlusal harmony and stability are maintained.

The concept of cuspid-protected occlusion, introduced and popularized by D'Amico, was employed due to its advantages over group function. This occlusal scheme helps direct forces along the long axis of teeth and minimizes harmful lateral stresses on posterior teeth. This emphasizes that canine guidance with immediate posterior disocclusion is a natural protective mechanism that reduces the risk of occlusal trauma and contributes to long-term oral health [11].

Additionally, studies by Hobo and Takayama demonstrated that among the determinants of occlusion-condylar path, incisal path, and cusp angle, the cusp angle is the most reliable and controllable factor [7,8]. Based on this principle, their twin-stage technique was adopted in this case. This method allows predictable control of disocclusion without the need to record condylar guidance, thereby simplifying the clinical procedure while maintaining precision.

Using this technique, standardized disocclusion values were achieved: approximately 1.1 mm in protrusive movements, 1 mm on the non-working side, and 0.5 mm on the working side during eccentric movements at 3 mm protrusion from centric relation. These parameters ensure optimal functional efficiency, occlusal harmony, and long-term stability of the prosthetic rehabilitation.

## Conclusions

Full-mouth rehabilitation in AI patients is complex but can be successfully managed using a systematic approach. The Hobo twin-stage technique provides a predictable method for restoring occlusion without complex recordings. In this case, functional efficiency, esthetics, and patient comfort were significantly improved. Long-term success depends on proper planning, execution, and follow-up.
